# Preoperative Virtual Reality to Expose Patients With Breast Cancer to the Operating Room Environment: Feasibility and Pilot Case Series Study

**DOI:** 10.2196/46367

**Published:** 2024-01-17

**Authors:** Jordana L Sommer, Kristin Reynolds, Pamela Hebbard, Michael S D Smith, Natalie Mota, W Alan C Mutch, Jessica Maples-Keller, Leslie Roos, Renée El-Gabalawy

**Affiliations:** 1 Department of Anesthesiology, Perioperative and Pain Medicine University of Manitoba Winnipeg, MB Canada; 2 Department of Psychology University of Manitoba Winnipeg, MB Canada; 3 Department of Psychiatry University of Manitoba Winnipeg, MB Canada; 4 Department of Surgery University of Manitoba Winnipeg, MB Canada; 5 CancerCare Manitoba Winnipeg, MB Canada; 6 National Research Council Canada Winnipeg, MB Canada; 7 Department of Clinical Health Psychology University of Manitoba Winnipeg, MB Canada; 8 Department of Psychiatry and Behavioral Sciences Emory University Atlanta, GA United States

**Keywords:** virtual reality, preoperative anxiety and distress, breast cancer surgery, anesthesia, feasibility, pilot

## Abstract

**Background:**

Clinically elevated preoperative distress and anxiety are common among patients undergoing cancer surgery. Preoperative interventions have been developed to mitigate this distress and anxiety but are inconsistent in efficacy and feasibility for broad implementation.

**Objective:**

This preliminary pilot study aims to assess the feasibility and utility of a newly developed virtual reality (VR) intervention to expose patients awaiting breast cancer surgery to the operating room environment and a simulation of anesthetic induction.

**Methods:**

Patients undergoing breast cancer surgery (N=7) were assigned to the VR intervention or control (treatment as usual) group and completed self-report measures of distress and anxiety before surgery, on the day of surgery, and after surgery (5 and 30 d postoperatively). Those in the intervention group trialed the VR simulation 1 to 2 weeks preoperatively and provided qualitative and quantitative feedback. We assessed the feasibility of recruitment capability and study design and evaluated participants’ impressions of the intervention using self-report rating scales and open-ended questions. We also descriptively examined distress and anxiety levels throughout the duration of the study.

**Results:**

Recruitment occurred between December 2021 and December 2022 and progressed slowly (rate: 1 participant/7 wk on average; some hesitancy because of stress and being overwhelmed). All participants who consented to participate completed the entire study. All participants were female and aged 56 (SD 10.56) years on average. In total, 57% (4/7) of the participants were assigned to the intervention group. On average, intervention participants spent 12 minutes engaged in the VR simulation. In general, the intervention was rated favorably (eg, clear information, enjoyable, and attractive presentation; mean*_% agreement_* 95.00-96.25, SD 4.79-10.00) and as helpful (mean*_% agreement_* 87.50, SD 25.00). Participants described the intervention as realistic (eg, “It was realistic to my past surgical experiences”), impacting their degree of preparedness and expectations for surgery (eg, “The sounds and sights and procedures give you a test run; they prepare you for the actual day”), and having a calming or relaxing effect (eg, “You feel more relaxed for the surgery”).

**Conclusions:**

This preoperative VR intervention demonstrated preliminary feasibility among a sample of patients undergoing breast cancer surgery. Results and participant feedback will inform modifications to the VR intervention and the study design of a large-scale randomized controlled trial to examine the efficacy of this intervention.

**Trial Registration:**

ClinicalTrials.gov NCT04544618; https://clinicaltrials.gov/study/NCT04544618

## Introduction

### Preoperative Distress

Preoperative distress, a situational emotional reaction (eg, fear, worry, and helplessness), is common among surgical patients [1,2]. Extant research has identified several adverse health-related perioperative outcomes of preoperative distress in both the presence and absence of a mental disorder [3-7]. In particular, patients undergoing cancer surgery experience clinically meaningful elevated rates of preoperative distress, ranging from 23% to 77% in recent research [8-10]. Preoperative distress is also associated with various adverse health-related outcomes for patients undergoing cancer surgery specifically, including increased postoperative pain, nausea, discomfort, and fatigue, among others [11-15]. In recognition of its detrimental impact, the National Comprehensive Cancer Network (NCCN) designated distress as the “6th vital sign” [16].

### Preoperative Psychological Interventions

Receiving a cancer diagnosis is a significant and life-altering event, often intensified by the necessity for major surgical intervention and an uncertain health trajectory. In considering the adverse health implications of psychological distress, several preoperative interventions (eg, education, relaxation training, and stress management) have been developed that seek to improve psychological and physical functioning before surgery by establishing realistic expectations of the surgical process and helping patients cope with surgery-related uncertainty and distress [17-24]. However, the literature reveals conflicting evidence regarding the efficacy of many such interventions [17-23]. Importantly, the interventions that are supported by evidence require delivery by licensed health care providers [21,22] and often require multiple sessions, rendering them impractical for large-scale implementation, particularly within the constraints of a publicly funded health care system.

### Virtual Reality Interventions

Virtual reality (VR) interventions have shown considerable promise in reducing psychological distress in nonsurgical contexts [25-30]. Research in this area has examined the effectiveness of VR exposure therapy for the treatment of anxiety and posttraumatic stress disorder [25,27,28,31-33], where the user virtually and systematically confronts feared content to overcome anxiety. Patients often prefer using VR for exposure therapy over traditional in vivo exposure [34,35], and it may also be more straightforward to administer. This innovative technology has also been gaining popularity in broader medical contexts and has shown promising results in pain management [36-40] and cognitive and physical rehabilitation among various medical populations [37,41-43]. In contrast to therapist-guided VR exposure used in mental health settings, which may be used as a component of one-on-one psychotherapy over a duration of months, VR interventions developed for use in medical settings do not typically require a specialized health care professional to administer and can often achieve desirable outcomes following a shorter duration of use [37,44-47].

### Preparatory Interventions for Stress Exposure

In a preoperative context, VR could be used to psychologically intervene before patients develop clinically elevated distress and are affected by the adverse downstream effects of distress (while also targeting any existing distress about surgery). This is similar to stress inoculation training, a form of cognitive behavioral intervention, aimed at psychologically preparing individuals for future exposure to a stressful environment through preliminary exposure to elements of that environment [48]. This form of intervention has been adapted using VR technology [48-52], and preliminary evidence supports reductions in predeployment distress for military personnel using such interventions to prepare for combat [50,53,54]. In fact, similar methods have been applied to psychologically prepare patients before surgery, including operating room (OR) tours before surgery [55], given that the OR environment is noted as distressing for many surgical patients [3,56,57]. Although this intervention was associated with reductions in preoperative distress [55], it has limited feasibility for broad administration because of the infrequent availability of ORs for such purposes and the limited resources and personnel to implement this intervention.

### Preoperative Applications of VR

The use of VR to expose patients to the OR environment and preoperative process resolves some of these limitations. A few studies have implemented such interventions to target preoperative distress and other perioperative outcomes, largely among pediatric patients (all but one of the identified studies) undergoing variable types of surgeries (eg, general, neurological, and plastics or ear, nose, and throat) [58-64]. Small-scale meta-analyses examining this literature support the initial efficacy of such interventions in reducing preoperative distress [65-67], although some studies have used VR distraction interventions (eg, using games or relaxation) as opposed to exposing users to the OR environment. Importantly, this research is in its infancy, and only a few studies exist in this area to date, supporting the need for further exploration.

### Gaps in the Literature

Although the preoperative VR interventions described in the preceding section demonstrate preliminary efficacy in mitigating preoperative distress and potential for broad implementation within the constraints of our health care system (ie, relatively low cost, do not require specialized professional training to administer, can be used repeatedly in different settings, translated into multiple languages, and adapted across surgery types), studies examining these interventions are not without limitations. First, most studies in this area have focused on samples of pediatric patients undergoing surgery; further research is needed to establish the efficacy of such interventions among adult patient samples. Second, no identified studies to date have evaluated a preoperative VR intervention using patients scheduled to undergo an oncological procedure, a population with elevated levels of preoperative distress [8-10]. Third, existing preoperative VR interventions have limited immersion capabilities (eg, lack of user embodiment [ie, the ability to visualize and manipulate virtual representations of the user’s body] and use of prerecorded virtual videos as opposed to a fully immersive virtual environment), which may weaken their impact on mitigating distress through reduced realism. Fourth, these studies lacked follow-up data beyond the acute postoperative phase (eg, <1 wk after discharge), which is needed to understand the long-term impact on postoperative recovery. Finally, many of these studies did not gather user feedback on the intervention, which is vital to help maximize the potential impacts of these interventions.

### This Study

In light of these identified gaps, this study aims to assess the feasibility of, and preliminarily pilot (in case series format), a novel VR OR simulation targeting preoperative distress and anxiety among a sample of patients undergoing breast cancer surgery. Specifically, regarding feasibility, the aims are to assess recruitment capability and identify resulting sample characteristics, understand participants’ impressions of the study design and intervention, and evaluate data collection procedures and outcome measures. Finally, this study will also pilot-test the preliminary impact of the intervention on perioperative distress and anxiety in a case series format. The results of this study will inform modifications to the VR simulation and the design of a large-scale randomized controlled trial (RCT) to evaluate the efficacy of this intervention.

## Methods

### Overview

This study used a single-blind randomized design to assess the feasibility of and pilot the VR simulation to expose patients undergoing breast cancer surgery to the OR and preoperative process. This study represents an in-depth preliminary analysis of a larger pilot study (ClinicalTrials.gov; NCT04544618). Participants were assigned to the VR intervention group or the treatment-as-usual (control) group at the time of recruitment. Randomization was stratified according to the type of breast cancer surgery (with vs without reconstruction) and whether neoadjuvant chemotherapy was received to enable equal proportions of participants with these characteristics across study groups; research demonstrates differences in distress levels according to these factors [68]. All participants completed self-report measures 1 to 2 weeks before surgery (ie, baseline; VR group participants tested the intervention at this time), on the day of surgery, 5 days after surgery, and 30 days after surgery. Notably, the initial planned design included a third study arm (ie, non–surgery-related VR; Nature Treks), which was ultimately dropped because of recruitment challenges. Ethical amendments were approved supporting this change (and others noted in the *Recruitment Capability and Sample Characteristics* section), and the trial registry has been updated accordingly.

### Participants

We recruited a convenience sample of adult patients undergoing breast cancer surgery by describing the study at patients’ surgical oncology appointments and preoperative information sessions and circulating study posters. Interested patients provided their contact information to enable a telephone discussion with the research coordinator and eligibility screening (those viewing the poster contacted the coordinator directly). Participants were eligible if they were being scheduled to undergo breast cancer surgery under general anesthesia at the Health Sciences Centre (a tertiary care hospital in Winnipeg, Canada) and could speak and read English. Those unable to provide informed consent or unable to participate in a VR intervention (eg, owing to visual or auditory impairment) were excluded. Our initial target was to recruit 15 participants per group, with a study aim to evaluate recruitment capability.

### Procedure

Participants randomized to the VR group trialed the intervention 1 to 2 weeks before surgery (baseline). Those in the control group received no additional intervention beyond their standard medical appointments and optional preoperative information sessions (offered to all patients). All participants completed self-report measures at baseline (those in the VR group received additional measures to assess intervention feedback). On the day of surgery, preoperative distress and anxiety were reassessed while the participants were in the preoperative holding area and again in the OR before anesthetic induction. At 5 days and 30 days after the operation, all participants were readministered a subset of the baseline measures, and those in the VR group provided additional intervention feedback at the 5-day postoperative assessment. The participants in the VR group completed baseline measures in person (at the time of the intervention), and all participants completed the day-of-surgery measures in person. All other measures were completed online through the web-based survey platform, Qualtrics (Qualtrics International Inc).

### Intervention

A VR development team at the National Research Council of Canada, in collaboration with coauthors (RE and JLS), developed the VR OR simulation for use in this research (a technical paper describing the simulation more in depth is in progress). The simulation development stages included creating an initial prototype based on the observation of surgeries and consultation with medical personnel, developing an anesthetic induction script based on example scripts provided by several anesthesiologists, integrating input from an anesthesiologist (WACM) on the initial prototype, and refining the platform through multiple iterations of feedback from coauthors. The VR simulation begins with the participant sitting on an examination table (reflected as the OR table in the simulation), wearing the VR head-mounted display, and holding the controllers (enabling user embodiment and visualization and manipulation of virtual arms). The participant is instructed to imagine it is their day of surgery, including how they might be feeling that day. The participant then spends at least 5 minutes exploring the virtual OR, which includes relevant machinery and equipment, personnel, sounds, and details such as a mammogram displayed on a computer screen. This free exploration is followed by a scripted portion, where the participant is instructed to lie supine on the virtual OR table and is taken through a mock anesthetic induction process led by the virtual anesthesiologist and nurse; the patient is prompted to answer questions similar to those they will be asked on the day of surgery (eg, name, date of birth, type of surgery, and allergies) and is virtually prepared with monitoring devices by the nurse (eg, blood pressure cuff, a pulse oximeter, and electrocardiogram stickers and electrodes). The simulation ends after the virtual oxygen mask is placed on the patient’s mouth and the screen darkens (refer to Multimedia Appendix 1). We used the Oculus Rift S VR system (Meta Platforms) with a cable connection to a laptop computer for the intervention administration.

### Ethical Considerations

This study was approved by the University of Manitoba Health Research Ethics Board (#HS23957). All participants provided written informed consent before participation. No participant-identifying information was included with the study data. Each participant was assigned a study identification number, which was used to collate participant data over the study duration. All participants were provided with a CAD $25 (US $18.94) gift card after completing the study, and the cost of parking for those attending an intervention appointment was reimbursed.

### Measures

#### Preoperative Distress

A total of 4 self-report measures assessed preoperative distress, including 2 preoperative-specific scales (Preoperative Intrusive Thoughts Inventory [PITI] and Amsterdam Preoperative Anxiety Information Scale [APAIS]) and 2 nonspecific visual analog scales (NCCN Distress Thermometer and adapted “Anxiety Thermometer”). The PITI and APAIS were only administered at baseline and on the day of surgery, whereas the Distress and Anxiety Thermometers were assessed at all 4 time points (and in the OR before induction). At the 5-day postoperative follow-up, the participants were asked which measure best captured their experience of distress or anxiety, which will inform the selection of the primary outcome measure for the upcoming RCT. At 5-day postoperative follow-up, they were also prompted to retrospectively rate their level of distress/anxiety from 0 (*no distress/anxiety*) to 10 (*extreme distress/anxiety*) corresponding to 8 different “events” ranging from prediagnosis (average level of distress/anxiety before receiving a cancer diagnosis) until the 5-day follow-up.

The PITI is a validated and reliable 20-item self-report measure of preoperative anxiety [69]. Items (eg, “I worry that I won’t wake up*”*) are rated on a 4-point scale, ranging from 0 (*not at all*) to 3 (*most of the time*). Summing the items yields a total score ranging from 0 to 60, where a score ≥15 indicates clinically significant preoperative anxiety [69]. The APAIS is a validated and reliable 6-item measure of preoperative anxiety [70]. Items (eg, “I am worried about the procedure”) are rated on a 5-point scale, ranging from 1 (*not at all*) to 5 (*extremely*). A total score is calculated by summing all items, ranging from 6 to 30, where a score ≥10 indicates clinically elevated preoperative anxiety [70]. The NCCN Distress Thermometer is a visual analog scale that resembles a thermometer, with a scale ranging from 0 (*no distress*) to 10 (*extreme distress*) [71]. This has been validated among several oncology samples [72,73]. Distress is rated using a “past-week” timeframe (modified to present time in the OR), and a cut-off score of 4 indicates clinically elevated distress [73]. Because of the common interchangeable use of the terms distress and anxiety within the perioperative and oncology literature and the lack of clear differentiation between these terms, we adapted the Distress Thermometer to create an Anxiety Thermometer.

#### VR Impressions and Feedback

Participants provided self-reported feedback on the VR simulation at 2 different time points. Feedback measures were developed in accordance with previous research [74]. After trialing the intervention, the participants were provided with a list of statements about their experience using the simulation (eg, “I found the VR intervention was helpful”), which they rated from 0% (*completely disagree*) to 100% (*completely agree*). The participants are also asked whether they experienced any motion sickness during the intervention (0 [*none*] to 3 [*severe*]), followed by open-ended questions prompting intervention impressions (eg, what they liked or disliked) and whether they found the intervention worthwhile. Finally, the participants were asked about additional elements they wished to be included in the intervention and were offered multiple response options for selection (eg, being wheeled into the OR). At the 5-day postoperative follow-up, the VR participants provided additional intervention feedback (eg, overall impressions). The participants are then asked if or how they think the intervention impacted their surgery or recovery, whether they disliked anything about it, and if they have any other suggestions for improvements. The participants are prompted to elaborate on their responses to these questions.

#### Presence

The iGroup Presence Questionnaire [75] assessed the presence associated with the VR intervention at baseline, defined as the sense of *being* in the virtual environment. This valid and reliable (Cronbach α=.87) self-report measure is comprised of 14 items (eg, “I had a sense of acting in the virtual space, rather than operating something from outside”), which are rated on a 7-point scale (−3 [*fully disagree*] to +3 [*fully agree*]). Items are summed to create 3 subscale scores (spatial presence, involvement, and realism), where higher scores indicate increased presence in the virtual environment.

#### Sample Characteristics

Participants self-reported their sociodemographic characteristics and health history at baseline, including age (assessed continuously), sex (female or male), marital status (single, married or common law, divorced or separated, or widowed), highest level of education (high school or less or some college or higher), stage of breast cancer, type of breast cancer surgery (eg, lumpectomy or single or double mastectomy with or without reconstruction), whether they are receiving chemotherapy before surgery, history of prior surgeries, mental health service use since receiving their cancer diagnosis, and history of receiving a mental health diagnosis. Various other self-report measures were administered throughout the study (eg, assessing depression, coping, and quality of life) to determine their feasibility for inclusion in the upcoming RCT (by calculating the proportion of missing data).

### Analytic Strategy

Descriptive statistics assessed the consent rate, recruitment speed, attrition rate, and sample characteristics. We calculated the participation rate among the intervention and control groups, and we assessed quantitative and qualitative intervention feedback descriptively. We then calculated the proportion of missing data, and descriptive statistics determined which measure of preoperative distress or anxiety was rated most favorably. Finally, we presented participants’ levels of distress and anxiety across the perioperative period (baseline to 30-day follow-up) descriptively in a case series format.

## Results

### Feasibility Aims

#### Recruitment Capability and Sample Characteristics

Recruitment was initiated on December 1, 2021. Between initiation and December 1, 2022, a total of 14 prospective participants were identified (n=5, 36% were identified in the final 2 months of recruitment). Of these 14 individuals, 12 (86%) contacted the study coordinator directly, 1 (7%) had their information provided by a health professional, and 1 (7%) expressed interest while attending a preoperative information session (in-person sessions were suspended until November 2022). Of these 14 individuals, 7 (50%) consented and participated, 4 (29%) were ineligible (eg, required to isolate until surgery or already had surgery), and 3 (21%) withdrew after providing verbal consent but before providing written consent (reasons: n=1, 33% too many appointments and unable to focus on anything else; n=1, 33% unwilling to come in person to try the VR; and n=1, 33% overwhelmed with family responsibilities; n=2, 67% had been randomized to the initial third arm before dropping out). The approximate recruitment speed for those who consented was 1 participant every 7 weeks, on average. In total, 57% (4/7) of the participants were assigned to the intervention group. Of those who provided written informed consent, 100% (7/7) completed the study. Because of ongoing recruitment challenges, the study target population was broadened 5 months after the initiation of recruitment to include any patients undergoing cancer surgery, as opposed to patients undergoing breast cancer surgery only. To date, no patients undergoing non–breast cancer surgery have expressed an interest in participating.

The participants were aged 56.43 (SD 10.56) years on average, and all were female. The participants were most commonly married (3/7, 43%), and the majority (5/7, 71%) had some college education or higher. The breast cancer stage of patients was most commonly uncertain or unknown (4/7, 57%). The most common surgical procedure was lumpectomy (4/7, 57%), and 43% (3/7) of the participants were planning to undergo reconstructive surgery. Most participants (6/7, 86%) had not received chemotherapy before their surgery, and most participants (6/7, 86%) had ≥1 prior surgeries. A total of 57% (4/7) of the participants reported receiving a mental health diagnosis in their lifetime (depression, anxiety, or substance use disorder), and 29% (2/7) of the participants indicated that they sought professional mental health support after receiving their cancer diagnosis. Most participants (4/7, 57% to 6/7, 86%) had clinically elevated preoperative distress or anxiety at baseline (Table 1).

**Table 1:** Sample characteristics for patients undergoing breast cancer surgery participating in the feasibility and pilot study to evaluate preoperative virtual reality (n=7).

Characteristic		Values
Age (y), mean (SD)		56.43 (10.56)
Sex (female), n (%)		7 (100)
**Marital status, n (%)**		
	Single	2 (29)
	Married or common law	3 (43)
	Divorced or separated	2 (29)
**Education, n (%)**		
	High school or less	2 (29)
	Some college or higher	5 (71)
**Stage of breast cancer, n (%)**		
	Uncertain (0-1, 1-2, or other unknown)	4 (57)
	Stage 1	1 (14)
	Stage 2	2 (29)
**Type of surgery, n (%)**		
	Lumpectomy	4 (57)
	Single mastectomy without reconstruction	1 (14)
	Singe mastectomy with immediate reconstruction	1 (14)
	Double mastectomy with immediate reconstruction	1 (14)
Undergoing reconstruction, n (%)		3 (43)
Neoadjuvant chemotherapy, n (%)		1 (14)
History of prior surgery, n (%)		6 (86)
Sought professional mental health support since cancer diagnosis, n (%)		2 (29)
Lifetime mental health diagnosis, n (%)		4 (57)
**Clinically significant preoperative distress or anxiety at baseline, n (%)**		
	PITI^a^	4 (57)
	APAIS^b^	6 (86)
	Distress thermometer	4 (57)
	Anxiety thermometer	5 (71)
Intervention group, n (%)		4 (57)

^a^PITI: Preoperative Intrusive Thoughts Inventory.

^b^APAIS: Amsterdam Preoperative Anxiety Information Scale.

#### Participant Impressions of the Study Design and Intervention

All participants assigned to the control group completed the entire study. All participants assigned to the intervention group, who provided written informed consent, tested the intervention within 2 weeks before their surgery and completed the study.

The participants in the intervention group spent 12 minutes engaged in the simulation, on average, and reported variable levels of presence while trialing the VR simulation (spatial presence: mean 8.75, involvement: mean 0.75, and realism: mean −2.50; refer to Table 2 for maximum ranges); the participants reported having a sense of being physically present in the virtual environment, with only partial attention devoted to the virtual environment, and moderate ratings of realism.

**Table 2:** Quantitative intervention impressions at baseline for patients undergoing breast cancer surgery assigned to the intervention group for the feasibility and pilot study evaluating preoperative virtual reality (VR; n=4).

Parameter		Values
VR duration (min), mean (SD)		11.64 (1.08)
Presence: spatial presence subscale (maximum range: −15 to 15), mean (SD)		8.75 (3.30)
Presence: involvement subscale (maximum range: −12 to 12), mean (SD)		0.75 (1.26)
Presence: realism subscale (maximum range: −15 to 15), mean (SD)		−2.50 (3.70)
The way information was presented was clear and understandable (0%-100%), mean (SD)		95.00 (10.00)
I enjoyed my session with the VR program (0%-100%), mean (SD)		96.25 (4.79)
I could understand and act on the information provided by the program (0%-100%), mean (SD)		93.75 (7.50)
The program had a very attractive presentation (0%-100%), mean (SD)		95.00 (5.77)
I had to look for assistance when I used this program (0%-100%), mean (SD)		42.50 (43.49)
The VR program froze or stopped unexpectedly (0%-100%), mean (SD)		5.00 (10.00)
I found the VR intervention was helpful (0%-100%), mean (SD)		87.50 (25.00)
The VR intervention eased my anxiety/concerns about the OR^a^ (0%-100%), mean (SD)		55.00 (47.96)
The VR intervention eased my anxiety/concerns about the anesthesia (0%-100%), mean (SD)		60.00 (45.46)
The VR intervention eased my anxiety/concerns about my surgery (0%-100%), mean (SD)		46.25 (38.16)
The VR intervention worsened my anxiety/concerns about the OR (0%-100%), mean (SD)		37.50 (47.87)
The VR intervention worsened my anxiety/concerns about the anesthesia (0%-100%), mean (SD)		25.00 (28.87)
The VR intervention worsened my anxiety/concerns about my surgery (0%-100%), mean (SD)		30.00 (51.96)
Experienced motion sickness, n (%)		0 (0)
Participating in the VR intervention was worthwhile considering time commitment, n (%)		4 (100)
**Other elements you would have liked to be included, n (%)**		
	Being wheeled into the OR	1 (25)
	Try on equipment (eg, oxygen mask) while engaged in the simulation	0 (0)
	Learn about the various machines I saw in the OR	1 (25)
	Ask the virtual anesthetist or nurse questions about my surgery	1 (25)
	None of the above	2 (50)
	Other (“real time pulse/heart rate”)	1 (25)

^a^OR: operating room.

There were minor technical difficulties for all 4 participants during the simulation (eg, difficulty finding the correct position lying down when prompted by the VR nurse), and the program needed to be restarted midway for 2 of the participants. In general, the participants found that the intervention presented information clearly, was enjoyable, easy to understand, and had an attractive presentation (mean_% agreement_ range: 93.75-96.25, SD range: 4.79-10.00). It was also generally rated as helpful (mean_% agreement_ 87.50, SD 25.00), and all participants considered participating in the VR intervention worthwhile considering the time commitment. The participants gave mixed ratings at baseline regarding the impact of the intervention on anxiety and concerns about the OR, anesthesia, and surgery. Given a list of suggestions for elements to be added to the intervention, a single participant selected each of the following: (1) being wheeled into the OR, (2) learn about the machines I saw in the OR, (3) ask the virtual anesthesiologist or nurse questions about surgery, and (4) other: “real time pulse/heart rate.”

Regarding open-ended feedback, multiple participants commented on the realism of the intervention, the impact of the intervention on expectation formation regarding surgery, and the calming or relaxing effect of the intervention (Textbox 1).

**Qualitative intervention impressions for patients undergoing breast cancer surgery assigned to the intervention group for the feasibility and pilot study evaluating preoperative virtual reality (VR; n=4).**

**Open-ended feedback (at baseline)**

- What did you like about the VR intervention
  “It is very realistic”
  “It was realistic to my past surgical experiences, it was interactive and could play a bit with it”
  “What to expect”
  “Just getting the feel of an OR” [operating room]
- What did you dislike about the VR intervention
  “Nothing”
  “Scary”
  “The program calibrated my body position a few times and had to be reset which is why I was more present in the real world than in the VR world”
  “Seemed like I was waiting for an hour until it told me to lie down”
- If you found it helpful: in what ways was the VR intervention helpful
  “The sounds and sights and procedures give you a test run- prepares you for the actual day”
  “Yes”
  “Was helpful in that it reminded me of all the noises, lights, and people necessary in an OR”
  “You feel more relaxed for the surgery”
- Explain why it was or was not worthwhile
  “Gave me information and made me think of my feelings, made me feel better”
  “I like to help with research and I’m curious about VR and mental health initiatives”
  “Knowing what to expect”
  “Think I can relax a bit now when it’s time for me to have my surgery”

**Assessed 5 d after surgery**

- Overall impression of the VR intervention
  “It was very good, very real to life. I liked it”
  “Head set didn’t work well”
  “I had past surgery and it was familiar from memory and with current surgery experience”
  “I thought it was a good way to help calm some of my fears”
- Elements from the OR that were missing from the VR which would have been helpful to include
  “No I think they covered everything”
  “If when they are putting stickers on etc. you would maybe lightly touch the spot”
  “Not that I remember. I wasn’t paying much attention to what everyone was doing or the equipment”
  “More condensed room, just focus on the 2 people in your face”
- Images or experiences from the VR intervention that stuck with you following the intervention
  “No”
  “The mask at the end”
  “The nurse moved in on my too quickly and startled me because she was so close so suddenly”
  “The lights”
- Components of in-hospital experiences on day of surgery that would have been helpful to include in VR
  “The waiting in the surgical admitting area. Sitting for a long time in a chair in the gown with IV pick in”
  “I didn’t get to wake up in the VR but it may be cool to wake up. You aren’t alone when they wake you up in case that unknown freaks people out”
  “The actual experience happened a lot quicker than the virtual experience. Speed up the simulation”
- How, if at all, do you think the VR simulation impacted your surgery or recovery
  “It makes a person more relaxed in the operating room”
  “Once they had me in the surgery room it was very fast”
  “If I didn’t have past experience then it would have helped me a lot but I was already familiar”
  “I believe it assisted me in that I was able to see the inside of an OR”
- Was there anything you disliked about the VR intervention (if yes, please describe)
  “No”
  “Sadly the program had to be reset a bunch of times because...the orientation was off. It brought me out of it”
  “It was way too long just sitting there and waiting for something to happen”
- Suggestions regarding how we can improve the VR simulation
  “No it was very informative”
  “Have the room smaller and things not so far away. People need to be closer to you”

#### Data Collection Procedures and Outcomes Measures

Across all the time points, only 0.7% of the data were missing. Most participants (5/7, 71%) reported that, of the different measures assessing anxiety and distress, the PITI best captured their experiences.

### Pilot Aim

#### Overview

Table 3 outlines the sample characteristics, and Table 4 outlines the perioperative levels of distress and anxiety of the participants in the control and intervention groups.

**Table 3:** Participant characteristics.

Participant ID	Group	Age (y)^a^	Current surgery	Prior surgery	Mental health history
P1	Control	60s	Lumpectomy	None	None
P2	Control	60s	Lumpectomy	Single mastectomy (>10 y ago)	Depression
P3	Control	40s	Double mastectomy with immediate reconstruction	Broken arm and appendectomy	None
P4	Intervention	60s	Lumpectomy	“Replacements” and “abnormal cell removals”	Mental health leave (no diagnosis)
P5	Intervention	40s	Single mastectomy with immediate reconstruction	Thyroid surgery >5 y ago	None
P6	Intervention	50s	Lumpectomy	Lumpectomy, fibroids removed, hysterectomy, cervix and ovaries removed, and deviated septum repair	Depression and anxiety
P7	Intervention	50s	Single mastectomy without reconstruction	Arm and cesarean section	Depression and substance use disorder

^a^Age range.

**Table 4:** Perioperative distress or anxiety for patients ongoing breast cancer surgery participating in the feasibility and pilot study to evaluate preoperative virtual reality.

Participant ID, group, and measure			Perioperative distress or anxiety^a^													Clinically elevated^b^
			Baseline		Preoperative		OR^c^		5 d postoperative		30 d postoperative					
**P1: control group**																
	PITI^d^ total	13.00		7.00		N/A^e^		N/A		N/A			0/2 (0)			
	APAIS^f^ total	10.00		12.00		N/A		N/A		N/A			2/2 (100)			
	Distress thermometer	0.00		0.00		0.00		2.00		2.00			0/5 (0)			
	Anxiety thermometer	0.00		0.00		0.00		2.00		2.00			0/5 (0)			
**P2: control group**																
	PITI total	26.00		32.00		N/A		N/A		N/A			2/2 (100)			
	APAIS total	13.00		18.00		N/A		N/A		N/A			2/2 (100)			
	Distress thermometer	3.00		6.50		8.00		2.00		5.00			3/5 (60)			
	Anxiety thermometer	3.00		6.50		5.00		3.00		3.00			2/5 (40)			
**P3: control group**																
	PITI total	42.00		37.00		N/A		N/A		N/A			2/2 (100)			
	APAIS total	19.00		17.00		N/A		N/A		N/A			2/2 (100)			
	Distress thermometer	8.00		9.00		7.00		8.00		8.00			5/5 (100)			
	Anxiety thermometer	9.00		9.00		8.00		6.00		9.00			5/5 (100)			
**P4: intervention group**																
	PITI total	10.00		12.00		N/A		N/A		N/A			0/2 (0)			
	APAIS total	11.00		8.00		N/A		N/A		N/A			1/2 (50)			
	Distress thermometer	6.00		3.00		1.00		6.00		4.00			3/5 (60)			
	Anxiety thermometer	6.00		3.00		1.00		3.00		5.00			2/5 (40)			
**P5: intervention group**																
	PITI total	47.00		40.00		N/A		N/A		N/A			2/2 (100)			
	APAIS total	21.00		24.00		N/A		N/A		N/A			2/2 (100)			
	Distress thermometer	10.00		10.00		—^g^		5.00		10.00			4/4 (100)			
	Anxiety thermometer	10.00		9.00		—		3.00		9.00			3/4 (75)			
**P6: intervention group**																
	PITI total	11.00		10.00		N/A		N/A		N/A			0/2 (0)			
	APAIS total	9.00		10.00		N/A		N/A		N/A			1/2 (50)			
	Distress thermometer	3.00		2.00		3.00		0.00		0.00			0/5 (0)			
	Anxiety thermometer	4.00		3.00		5.00		2.00		4.00			3/5 (60)			
**P7: intervention group**																
	PITI total	43.00		49.00		N/A		N/A		N/A			2/2 (100)			
	APAIS total	21.00		22.00		N/A		N/A		N/A			2/2 (100)			
	Distress thermometer	7.00		8.00		9.00		7.00		4.00			5/5 (100)			
	Anxiety thermometer	7.00		9.00		10.00		6.00		5.00			5/5 (100)			

^a^Values represent total scores on each measure at each time point.

^b^Values represent the number of times a score is clinically elevated across the total number of measurements.

^c^OR: operating room.

^d^PITI: Preoperative Intrusive Thoughts Inventory.

^e^N/A: not applicable; PITI and Amsterdam Preoperative Anxiety Information Scale are specific to the preoperative period and were not administered in the OR or during the postoperative phase.

^f^APAIS: Amsterdam Preoperative Anxiety Information Scale.

^g^Missing data because of surgery scheduling change.

#### Retrospective Reports of Distress or Anxiety

**Figure 1:**
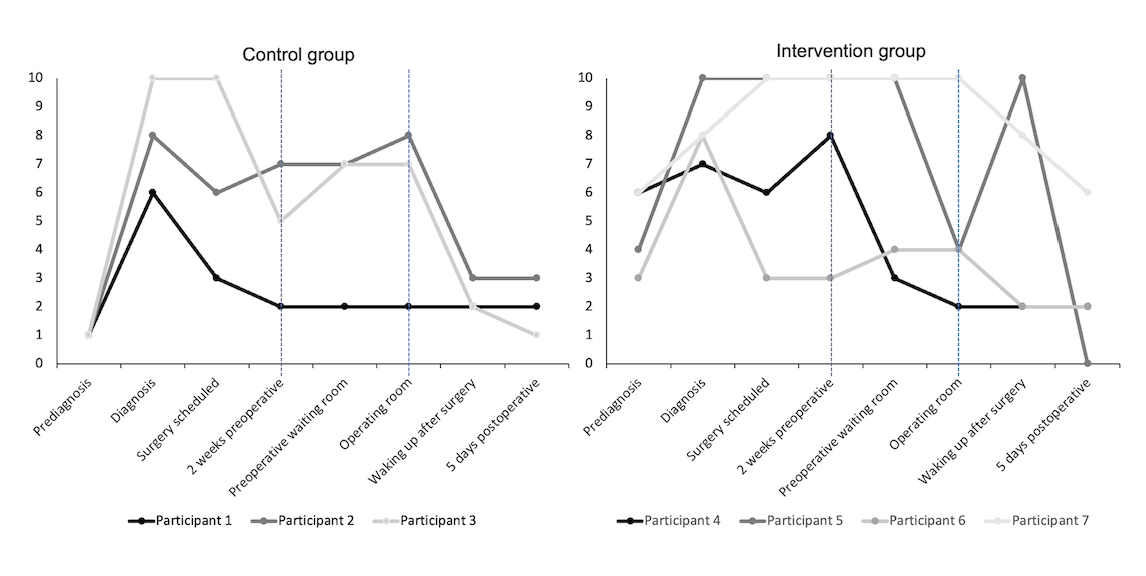
Retrospective reports of distress/anxiety among the control group and intervention group for patients undergoing breast cancer surgery participating in the feasibility and pilot study to evaluate preoperative virtual reality (VR). Blue guidelines outline the period between when the intervention group trialed the VR and participants’ day of surgery.

As shown in Figure 1, among the control group, ratings of distress/anxiety remained stable (P1) or increased (P2 and P3) between baseline (within 2-wk preoperatively) and being in the OR on the day of surgery. Among the intervention group, ratings decreased between baseline (within 2-wk preoperatively; when VR was administered) and being in the OR for 50% (2/4; P4 and P5) of the participants.

## Discussion

### Overview

To our knowledge, this is the first study to date to examine the feasibility of, and preliminarily pilot, a novel preoperative VR intervention exposing patients undergoing breast cancer surgery to the OR and preoperative process. Overall, despite some recruitment challenges, the intervention was generally rated favorably and described, on average, as 87.5% (SD 25.00%) helpful by participants. The results of this study will inform modifications made to the VR intervention and the study design of an upcoming RCT evaluating this intervention.

The newly developed VR intervention exposed patients undergoing breast cancer surgery to the OR environment (including machinery, sounds, personnel, and other medical items [eg, surgical tools and mammogram]) and preoperative process (from being seated on the OR table until completion of anesthetic induction). The simulation was developed to mimic the real-life OR and preoperative experience based on a large tertiary care hospital in Winnipeg, Manitoba. Compared with other recently developed preoperative VR interventions [58-64], this was designed to be more immersive through the integration of user embodiment (including visualization of one’s virtual body and real-time manipulation of virtual arms) and is one of the few interventions designed for adult use and the only such intervention developed and tested in Canada.

Although some technical difficulties arose during the intervention (eg, simulation needing to be restarted and slight delay in progression because of imprecise positioning of participant arm or body), likely detracting from immersion, the participants described the intervention as realistic and commented on its impact on feeling more prepared or knowing what to expect for surgery and feeling more relaxed or calm about their upcoming surgery. The participants also rated the intervention favorably in terms of enjoyment, clarity of information, attractiveness, and helpfulness. Although the sample size of this study limits our ability to establish trends regarding the impact of the intervention on distress and anxiety, the participants rated 46% to 60% agreement (SD range 38.16%-47.96%), on average, that the intervention eased their anxiety, and for half of the intervention group participants (2/4, 50%), retrospective ratings of distress/anxiety declined between trialing the intervention and being in the OR. Notably, the participants also rated 25% to 38% (SD range 28.87%-51.96%) agreement, on average, that the intervention worsened their anxiety (immediately postintervention), although they did not indicate this when providing feedback postoperatively. This may suggest the activation of the “fear structure” within the simulation, which is noted as an important component of anxiety-based exposure interventions [76].

Although preliminary data support the feasibility of the VR intervention, we encountered challenges regarding recruitment for the study. This may have been impacted by various factors including changes to surgical scheduling during the COVID-19 pandemic (noted in recent research on patients with cancer [77]), prospective participants’ reported feelings of being overwhelmed and stressed by their own health or other commitments, and a strained health care system resulting in reduced resources to support recruitment (including canceling in-person preoperative information sessions for 10 months during the recruitment period, where recruitment was planned to take place). As noted, recruitment began improving over the final 2 months of the recruitment period, wherein 80% (4/5) of the individuals who expressed interest in the study provided consent to participate. Although speculative, this may suggest an impact of the changing centrality of the pandemic on recruitment capability. Interestingly, most participants (6/7, 86%) had a history of prior surgeries, which could have resulted in an increased willingness to participate. It may be worthwhile to consider modifications to our recruitment poster (eg, including the rationale for the intervention) to entice participation from those who have not undergone prior surgery. The study design elements, including data collection, intervention engagement, and participant retention, appear feasible based on the current data.

### Strengths and Limitations

Despite the strengths of this study, including the novel preoperative VR intervention integrating user embodiment, evaluation of the feasibility of this intervention in a population with elevated estimates of clinically significant distress [8-10], collection of qualitative and quantitative intervention feedback, and inclusion of 2 iterations of postoperative follow-up data (5 and 30 days postoperatively; to be evaluated in an upcoming larger study), this study is not without limitations. First, recruitment challenges limited our sample size for this initial study; however, these challenges provided important information regarding the feasibility of implementing a larger study in the future. Second, there were a few technical difficulties encountered when administering the VR intervention, detracting from user immersion. Finally, although not directly investigated, distress in this population (and assessed using nonspecific measures) is likely to be influenced by many factors in addition to surgery. This particular intervention may not be very beneficial or impactful for those with primarily non–surgery-related distress.

### Implications

Importantly, these limitations, along with the data collected as part of this study, provide important insights to inform modifications to the intervention and study design before the implementation of a large-scale RCT to evaluate the efficacy of this intervention. Regarding recruitment, we will consider ways to target enhancing the involvement of health care professionals in spreading awareness of the study to potentially eligible patients while continuing to attempt recruitment at the newly reinstated in-person preoperative information sessions. In addition, we will consider including additional information about the intervention (and thus removing participant blinding) as part of the recruitment process. Changes to consider for the VR simulation include modifying requirements for the user’s body positioning to avoid unnecessary interruptions and potentially adding elements that participants noted would have been helpful (eg, the opportunity to learn about OR machines and ask questions to the virtual anesthetist or nurse). On the basis of participant feedback, this intervention has the potential to reduce levels of preoperative distress/anxiety by helping participants form more realistic expectations of the day of surgery before their operation (thus potentially reducing their perception of threat associated with the preoperative experience and enhancing their perceived ability to cope with this stressor). In line with recommendations based on other VR exposure-based interventions [78], having repeated exposure to the simulation may enhance the potential impact on mitigating distress/anxiety. Thus, it may be beneficial to assess the utility of providing participants with a 2D “screen-capture” video recording of their VR trial to watch on their own device multiple times in between trialing the intervention and their surgery. This may be an important avenue for future research evaluating this intervention.

Overall, this study established the initial feasibility of a novel preoperative VR intervention to expose patients undergoing breast cancer surgery to the OR and anesthetic induction process. These results will inform the study design of an upcoming large RCT to further examine this intervention. Participant feedback supports the utility and acceptability of this intervention and will inform future adaptations to the simulation. If demonstrated as efficacious in upcoming research, this intervention has the potential to be adapted across multiple surgery types and implemented on a broad scale to help mitigate preoperative distress.

## Appendix

Multimedia Appendix 1 Virtual reality simulation screen captures.

## Abbreviations

APAIS: Amsterdam Preoperative Anxiety Information Scale
NCCN: National Comprehensive Cancer Network
OR: operating room
PITI: Preoperative Intrusive Thoughts Inventory
RCT: randomized controlled trial
VR: virtual reality

## Data Availability

Data access is restricted to protect the confidentiality of participants and in light of ongoing research to expand the data set.
