# Supplementary figures and images for: Preoperative Virtual Reality to Expose Patients With Breast Cancer to the Operating Room Environment: Feasibility and Pilot Case Series Study
Source: JMIR Form Res. 2024 Jan 17;8:e46367. doi: 10.2196/46367 (PMC10831694; doi:10.2196/46367)

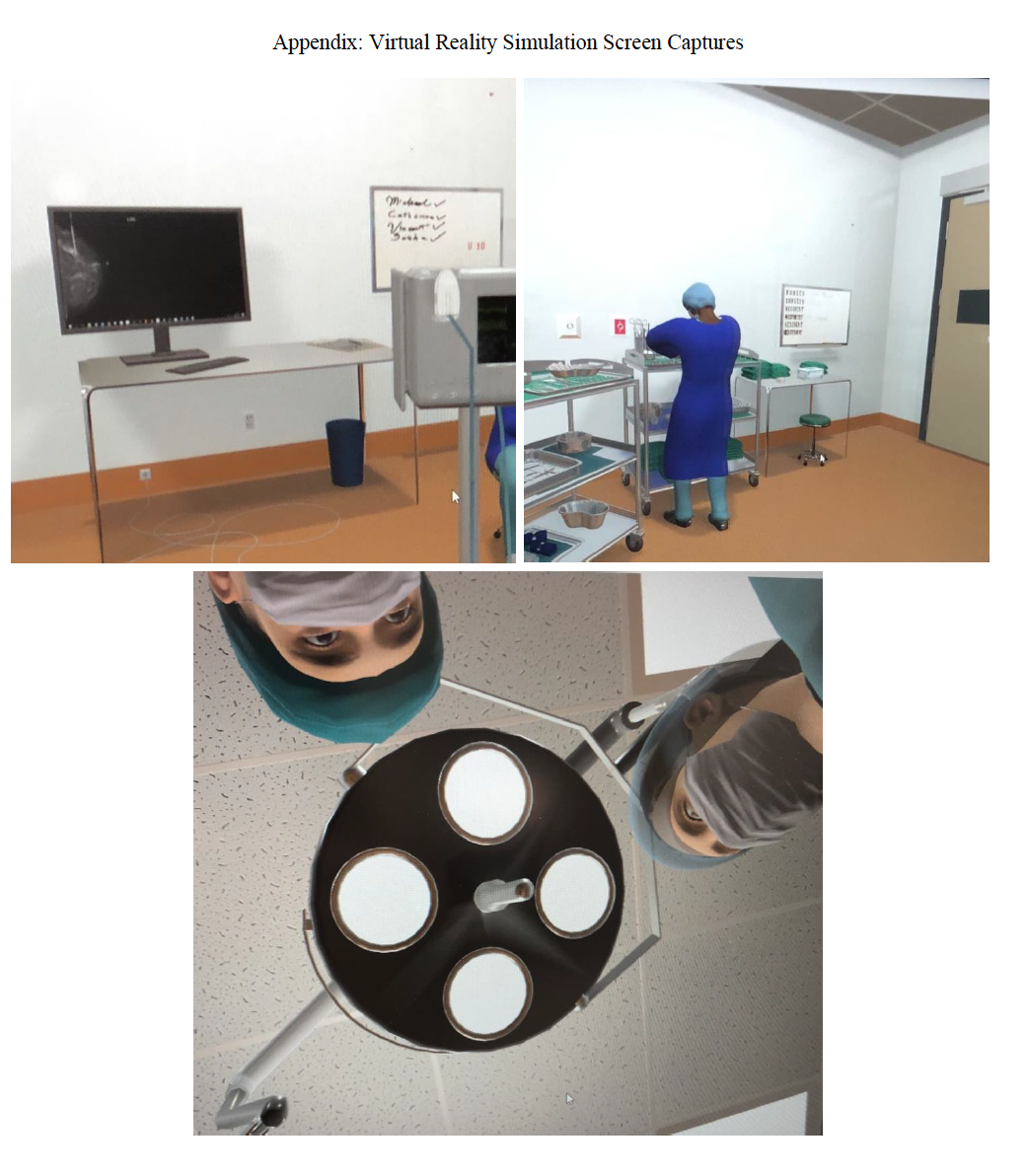

Supplement: Multimedia Appendix 1 [file formative_v8i1e46367_app1.png]
